# Reproductive health needs of HIV serodiscordant couples: a systematic review

**DOI:** 10.3389/fpubh.2024.1348026

**Published:** 2024-08-29

**Authors:** Mohadese Motaharinezhad, Zahra Yousefi, Sahar Rostami, Shahrbanoo Goli, Afsaneh Keramat

**Affiliations:** ^1^Student Research Committee, School of Nursing and Midwifery, Shahroud University of Medical Sciences, Shahroud, Iran; ^2^School of Allied Medical Sciences, Shahroud University of Medical Sciences, Shahroud, Iran; ^3^Department of Anatomy, School of Medicine, Tehran University of Medical Sciences, Tehran, Iran; ^4^Department of Infertility, Yas Hospital Complex, Tehran University of Medical Sciences, Tehran, Iran; ^5^Department of Epidemiology, School of Public Health, Shahroud University of Medical Sciences, Shahroud, Iran; ^6^Center for Health Related Social and Behavioral Sciences Research, Shahroud University of Medical Sciences, Shahroud, Iran

**Keywords:** HIV, serodiscordant, reproductive health, need, mix status

## Abstract

**Objectives:**

HIV is closely linked to reproductive and sexual health. HIV Serodiscordant couples face significant social, reproductive, and sexual challenges. This systematic review aimed to identify their reproductive health needs.

**Methods:**

A comprehensive literature search was conducted across six databases: Scopus, PubMed, Web of Science, Google Scholar, Magiran, and Iranmedex. No date restrictions were applied, and only English-language articles published before February 21, 2023, were included. We also searched the grey literature and conducted forward/backward citation searches.

**Results:**

From an initial 758 articles, 18 met the inclusion criteria. Studies were qualitative (*n* = 10) and quantitative (*n* = 8). Key reproductive health needs included (1) childbearing intention, (2) HIV serodiscordance and sexuality, (3) psychological and social support, (4) training and consultation services, (5) access to reliable information, and (6) focused training for healthcare providers.

**Conclusion:**

HIV-discordant couples face various reproductive health challenges. Implementing comprehensive guidelines for reproductive and sexual health, rehabilitation, and fertility planning is crucial to improving their quality of life and health.

**Systematic review registration:**

https://www.crd.york.ac.uk/prospero/, identifier CRD42023393567.

## 1 Introduction

Human immunodeficiency virus (HIV)-associated acquired immunodeficiency syndrome (AIDS), the second leading cause of death from infectious diseases globally (1), remains a significant public health concern (2). It has been estimated that 37.3 million people are affected by HIV/AIDS globally. Multiple biological, social, and psychological dimensions of HIV infection make it a highly complex health issue that impacts people’s quality of life (3). However, despite improvements in the health and well-being of HIV/AIDS patients, with HIV incidence stabilizing in most regions, the number of serodiscordant couples (discordant HIV status between partners) has risen (4). Estimates of HIV serodiscordant individuals vary from country to country (5). Globally, half of HIV-positive people are in long-term, regular relationships with HIV-negative partners (6). The development of new therapeutic approaches, particularly antiretroviral drugs, over the past decade has significantly improved the quality of life and life expectancy of seropositive individuals. This has enabled serodiscordant couples to consider pregnancy with greater confidence (7, 8). Despite the risk of perinatal and sexual transmission of HIV in HIV serodiscordant couples, they report maintaining their fertility goals and experiencing pregnancy rates similar to the general population (9). HIV is closely linked to reproductive and sexual health (10). Serodiscordant couples face a multitude of social, reproductive, and sexual challenges, often overlooked (11) This results in their being deprived of the dignity every citizen deserves due to the unfair social stigma attached to them as infected or immoral people (12). Empirical evidence shows that HIV-positive discordant couples are often neglected and are, at best, vaguely addressed in national prevention programs (13). The neglect of serodiscordant couples’ needs may stem from a combination of factors: the sensitive nature of HIV within couples, misconceptions regarding the degree of incompatibility, and a lack of comprehensive knowledge about available methods for preventing viral transmission (14). It is difficult to understand the issue of discordant couples regarding HIV/AIDS. Limited understanding of HIV management in serodiscordant couples is evident among both the couples themselves and healthcare professionals (15). While jurisprudence from international, regional, and national bodies reflects consideration of some health and human rights issues related to people living with HIV and SRH,^1^ the approach of these bodies has mainly been *ad-hoc* and lacks a systematic integration of human rights concerns of people living with HIV concerning SRH (16). The majority of studies on HIV serodiscordant couples have been performed in African countries. These studies mainly focus on issues such as perceptions of HIV risk, trust between partners, support received from male partners, communication problems, processes of decision-making, and HIV-related stigma (17, 18). High-quality reproductive and sexual healthcare services should be provided for HIV serodiscordant couples just like the general population. These populations have reproductive health needs that must be met, such as safe sexual intercourse, family planning, contraception, infertility treatments, and so on. Given the paucity of comprehensive reviews, this study will critically evaluate and synthesize the available evidence and current recommendations concerning the sexual and reproductive health needs of heterosexual serodiscordant couples. This study aimed to elucidate the scope of existing published literature, identify knowledge gaps, and propose recommendations for future research endeavors.

## 2 Methods

This study was approved by the Ethics Committee of Shahroud University of Medical Sciences (SHMU) (approval code: IR.SHMU.REC.1400.85) and prospectively registered with the International Prospective Register of Systematic Reviews (PROSPERO) (registration code: CRD42023393567). This systematic review adhered to the Preferred Reporting Items for Systematic Reviews and Meta-Analyses (PRISMA) guidelines throughout the review process.

### 2.1 Data sources

A comprehensive search was conducted across multiple medical databases, including SCOPUS, PubMed, Web of Science, and Google Scholar. Additionally, Iranian databases such as Magiran, the Scientific Information Database, and Iranmedex were searched. Open grey literature was assessed using Open Grey.^2^

### 2.2 Search strategy

Pre-selected Medical Subject Headings (MeSH) terms and text words were used and searched in the identified databases for peer-reviewed articles published before February 21, 2023. The search was limited to English-language publications. Search details, terms, and database-specific indexing terminology are available in the Supplementary material. We extracted all relevant studies by reading their titles, abstracts, keywords, and full texts.

### 2.3 Eligibility criteria

Studies investigating the reproductive health needs of serodiscordant couples were included. Standard elements of reproductive health are family planning services, counseling, information and education, prenatal care, safe delivery, postnatal care, infertility, STIs, counseling on sexuality and treatment of sexual dysfunction, etc. In our search, we selected articles that addressed a set of these needs, not just one. Excluded studies included: repeated data, insufficient data for analysis, unavailable full text, commentaries, opinion pieces, and editorials.

### 2.4 Data collection

After removing duplicates, the first author screened titles and abstracts of all retrieved records against the eligibility criteria. Initial screening involved reviewing titles and abstracts. Subsequently, full texts of relevant articles were retrieved for comprehensive assessment and final selection. The first author assessed each full-text article and extracted the required data, with the last author checking the extracted data. Any disagreements were resolved through discussion with a third reviewer. Corresponding authors of original studies were contacted for clarification or additional information, if necessary. Endnote was used to manage search results. Data extraction focused on researchers’ interpretations, categorized as themes, ideas, or concepts identified from the results and discussion sections to identify relevant information supported by the researchers’ interpretations of each paper. We further examined the discussion sections to identify relevant information supported by the researchers’ interpretations. A pre-designed data extraction form was used to collect information including manuscript authors, year of publication, country of origin, study design and method, sample size, and study results.

### 2.5 Assessment of risk of bias

A critical appraisal of the qualitative studies was conducted using the Critical Appraisal Skills Programme (CASP) checklist. Developed by the JAMA Group, CASP is a standardized tool for evaluating qualitative study methodology and data presentation (19, 20). The CASP checklist includes ten questions assessing the validity and accuracy of qualitative research. The majority of questions follow a three-point response format, with scores ranging from 0 to 2. A score of 2 indicates “yes,” 1 indicates “uncertain,” and 0 indicates “no”. Completion of the full checklist depends on the answers to the first two screening questions. If the answer to both is “yes,” the remaining questions could be assessed. Studies scoring a total of 20 were considered high-quality, 16–19 were considered medium-quality, and 15 or lower were considered low-quality.

One of the most well-known scales for assessing the quality and risk of bias in observational studies is the Newcastle-Ottawa Scale (NOS). The NOS checklist awards up to ten points in three domains: selection (0–5 points), comparability (0–2 points), and exposure/outcome (0–3 points). According to the NOS, the quality of the articles was rated in a range of 0–10. Studies were categorized as follows: perfect (9–10 points), good (7–8 points), satisfactory (5–6 points), and unsatisfactory (0–4 points) (21, 22).

Following a comprehensive assessment of each article’s full text, the first researcher completed the quality evaluation checklists. The same protocol was followed by the second researcher for re-assessment. In case of disagreement on the scoring of the items, the final score was taken in a joint meeting. In the subsequent step, the scores assigned to each domain and the overall scores were compared. Finally, studies were classified into three categories: good, medium, and poor-quality, based on the scores obtained from the checklist. The quality assessment yielded seven studies classified as good quality and one study classified as average quality.

### 2.6 Analysis

We reported study findings and employed a mixed-methods approach, conducting a qualitative and descriptive analysis based on the reported outcomes. Quantitative and qualitative findings were analyzed separately. The results of quantitative studies were descriptively reported by first creating an initial combination of conclusions, existing relationships, and examination of data strength. Thematic synthesis was used to analyze qualitative data. This involved a line-by-line examination of the results sections of the included studies, followed by the identification and discussion of descriptive themes. Thematic synthesis involves a three-stage process: initial line-by-line coding of the text, followed by the development of descriptive themes, and culminating in the generation of analytical themes. The thematic synthesis aimed to enhance understanding of questions regarding: “what works for whom and in what context”. We employed a thematic synthesis approach to analyze the findings. This involved initially extracting data from each primary study, followed by independent interpretation. Subsequently, we identified key themes across all studies to achieve a comprehensive analysis. Two reviewers independently coded key descriptive themes. We discussed the resulting themes and sub-themes within the study team as the analysis progressed to examine their relationship to the synthesis outcomes. The basic units of the review were elements of the texts reported in the “result” section of each primary study included in the analysis. Sections of the text were coded, with multiple codes allocated where appropriate. The qualitative synthesis then used the “descriptive themes” to develop “analytical themes”, which were interpreted about the synthesis aims. During the analysis, differences or similarities were identified within emerging themes.

## 3 Results

**Figure 1:**
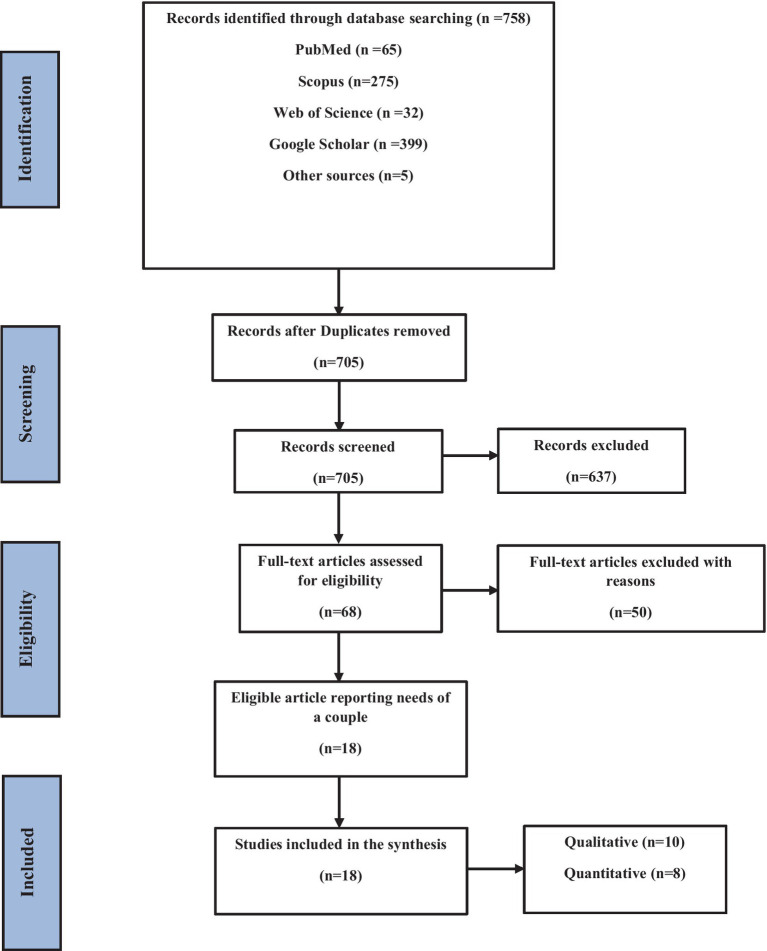
Flow diagram of study selection, based on PRISMA.

A total of 758 records were identified through the initial search, of which 705 eligible studies with available full-texts were selected (Figure 1). According to the review eligibility criteria, 637 papers were further excluded. Of these, 432 were excluded by title screening, leaving 205 abstracts to be reviewed. After that, 68 full-text publications were reviewed, and 50 of them were excluded. Finally, 18 articles were included in the synthesis. Studies were conducted in the United States of America (USA) (*N* = 6), Asia (*N* = 2) and Africa (*N* = 10). The studies were primarily qualitative (*N* = 10), and quantitative (*N* = 8). The characteristics of the studies are shown in Tables 1, 2.

**Table 1:** Characteristics of qualitative studies in the review.

No	Study, year (Reference)	Origin of the study	Study aim	Design and methodology	Summary of findings
1	Mahoney et al., 2015 (23)	America	To explore healthcare needs, perceptions of clinic-based prevention services, and experience of having an HIV-infected partner in HIV-negative partners.	10 seronegative participants participated in in-depth interviews. Eligible participants included those who tested HIV-negative within the last 6 months, had an HIV-positive sexual partner, and were aware of their partner’s HIV status.	Analysis of the interviews revealed some key themes: 1—Health needs during joint meetings Joint meetings were perceived as convenient and an opportunity to share health information as a couple. However, seronegative partners often struggled with their particular physical and mental health problems that were usually neglected during medical visits. 2—Strategies to reduce sexual risks Couples used a range of prevention strategies, including PrEP, mutual masturbation, sexual positions with lower risk of transmission, using condoms, and nonsexual intimacy. Several obstacles to condom use were reported. 3—Relationship dynamics Often, the relationship was characterized by mutual dependence, in which the HIV+ partner was both dependent and dominant. 4—Coping strategies: Adopting a mindful attitude, tapping into one’s spirituality, and reaching out to others.

2	Hughes et al., 2017 (24)	Brazil	Challenges faced by heterosexual couples of mixed HIV status [understand the place of HIV in the everyday lives of heterosexual mixed-status couples, how they constructed risk (including, but not exclusively, of HIV transmission) and managed their relationships]	Six couples were recruited in a two-step process. Data were collected over 11 months via participant observation sessions and repeated semi-structured interviews. The study design envisaged interviewing members of each couple both jointly and individually at least once. Data underwent thematic text analysis using both inductive and deductive approaches.	The needs and experiences of these couples varied depending on whether the partners became aware that one member was HIV-positive in the course of their relationship (sero-discovering) or both partners were aware of their serodiscordance from the outset (sero-cognisant). Because they include HIV testing, treatment, informational access, and support for both partners at some point (or points) during their relationship, these needs may seem similar. However, breaking down “serodiscordant couples” as a category reveals that these needs can occur at different times and with varying degrees of urgency.
3	Were et al., 2008 (25)	Kenya	To describe the perceptions of key stakeholders regarding the counseling the needs of HIV serodiscordant couples.	A purposive sample of nine key informants and 31 couple interviews totaling 71 participants. The couple interviews consisted of HIV untested, HIV concordant (positive and negative) and discordant couples	The following responses reported the most important issues in counseling HIV-discordant couples: Education about the HIV serodiscordant status and potential sources of infection Facilitating positive test results discloser to the negative partner. Discussion of the stigma surrounding formula feeding. Overall, participants supported safer sexual practices in discordant partnerships.
4	Larki et al., 2020 (13)	Iran	To explore the challenges faced by HIV-negative women in serodiscordant relationships.	15 HIV-negative women who were living with their HIV-positive husbands were selected through the purposive sampling method. Data were collected using semi-structured interviews. Data were analyzed using conventional content analysis adopted by Graneheim and Lundman.	“Threats to family life,” was the main theme, divided into five categories and their subcategories: Stigmatizing reactions after disclosure of the status. Social bise and scarcity of reliable information sources. Emotional disruptions. Difficult choices regarding fertility. In-family conflict over roles.
5	Kim et al., 2016 (26)	Uganda	To explore some of the factors involved in HIV transmission, maintaining discordant status, couples’ beliefs, and knowledge about serodiscordance and prevention practices.	In-depth interviews were conducted with 28 serodiscordant couples. NVivo Software Version 10 (QSR International, Victoria, Australia) was used for all coding and qualitative analysis.	Most couples had trouble describing the serodiscordance phenomenon and frequently struggled with prevention. Many people still believe in the pseudoscientific theories of blood type and blood “strength” as causes of HIV susceptibility/tolerance. The participants’ confidence in medical care and services was strong. Both serodiscordance and treatment were viewed more dimly and with mistrust of the communities. HIV-positive status and serodiscordance were frequently reported to be stigmatized.
6	Bunnell et al., 2005 (27)	Uganda	To explore explanations of and knowledge about discordance, challenges, and strategies for living with discordance	24 in-depth interviews and four focus group discussions were conducted using semi-structured guides.	Couple beliefs and knowledge about HIV serodiscordance: 1—Explanations for discordance: Most clients and counselors explained discordance by denying it was possible. A significant minority of clients believed that some people are immune from HIV infection. Clients in this study associated having a ‘strong’ blood composition with increased immunity. The infection can be transmitted only through “rough” sex. Defense against the disease from God: Some clients viewed their HIV-negative status, or that of their partner, as a reward from God 2—Explanations for challenges: Feelings of loneliness: Almost all counselors and couple members perceived HIV discordance to be rare, and, as a result, many HIV-discordant couples felt isolated by their test results. Relationships tension: In many couples, the initial reaction to their discordant results was to equate discordance with infidelity on the part of the HIV-positive partner. Difficulties in management of sexual relations: Sexual relations posed the most formidable challenge for couples, particularly women. Many HIV-positive clients stated that they had lost interest in sex entirely due to their discordant status. 3—Explanations for coping and prevention strategies: Using Condom: Condom use emerged as the most prevalent and preferred strategy for HIV prevention among discordant couples. Separation: Couples, particularly those in shorter relationships without children, also reported separation as a strategy to prevent HIV transmission. Abstinence: Although few couples had chosen abstinence as their coping strategy, many of the HIV-negative females said they would have preferred abstinence had their HIV-positive partners not refused.
				67 participants (32 women and 35 men). Both male and female members of 24 couples were interviewed. 15 discordant couples in which the male was HIV-positive, 9 discordant couples in which the female was HIV positive, 6 HIV-negative now single men, 6 HIV negative now single women, 4 HIV-positive now single men, and 2 HIV-positive now single women. Ethnography (version 4.0, Qualis Research Associates, Amherst, MA, USA) software was used to code and analyze the data.	
7	Crankshaw et al., 2014 (28)	South Africa	Comments and experiences of HCPs (physicians, nurses, and counselors) regarding safer conception.	Six semi-structured individual interviews per site (a total of 12 interviews), as well as a focus group discussion (FGD) at each site (a total of two focus groups). The FGDs included a total of 13 participants and consisted of seven nurses and six lay counselors working at the two sites.	The worriers of HCP are among the specific themes: Addressing serodiscordance Concerns about HIV disclosure Male partner participation
8	Langendorf et al., 2017 (29)	Brazil	To understand the meaning of pregnancy for heterosexual couples facing serodiscordant situations for HIV.	Phenomenological interview, with the participation of 11 couples The methodological framework of Martin Heidegger, which has two parts: (1) Vague Average Understanding and (2) Hermeneutics was used to analyze the data.	Unit of meaning: The significance of nursing care in the objective (biological, clinical) and subjective (social, existential) plans for serodiscordant heterosexual couples’ reproductive health Approaching care integrality Allowing for the protection of sexual and reproductive freedom
9	Wella, 2014 (30)	Malawi	To investigate how serodiscordant couples experience HIV and AIDS information.	Phenomenological in-depth interviews were used to invite serodiscordant couples to describe how they experience HIV and AIDS information. 21 serodiscordant couples (42 participants) 10 couples had the female partner HIV positive, and 11 had the male partner HIV positive. The interview data were analyzed using Van Manen’s approach to the analysis of phenomenological data.	The difficulties serodiscordant couples face in obtaining information on HIV and AIDS: Serodiscordance-related information was scarce and superficial The learning and information needs of the two partners differ HIV-negative partners are unable to attend meetings of HIV support group Illiteracy Disregarding libraries Language employed
10	Lelaka et al., 2022 (31)	South Africa	To examine the psychosocial support provided for HIV serodiscordant couples both in healthcare settings and in the community.	An interpretative phenomenological analysis (IPA) design was utilized for this study In-depth interviews were conducted with thirteen HIV serodiscordant couples. Data analysis was conducted using an interpretative phenomenological analysis framework.	Data analysis reported two superordinate themes, together with several categories or sub-themes on the support provided to participants in HIV serodiscordant relationships both at institutional and community level settings: 1: Assistance from institutions The assistance of service providers. 2: Assistance from the community level: Assistance from the family members Assistance from the partner

**Table 2:** Characteristics of quantitative studies in the review.

No	Study, year (Reference)	Origin of the study	Objectives	Design and methodology	Disclosure outcomes report	
1	Pottinger and Carroll, 2020 (32)	Jamaica	To examine fertility desire and motives for having children among PLHIV and explore the association with depressive symptoms.	Cross-sectional study 251 PLHIV in their reproductive years (18-49y) Gender: Male: 121 Female: 130 Age: < 40 years: 150 ≥ 40 years: 101 Relationship serostatus: Seroconcordant: 45 (18%) Serodiscordant: 65 (26%) Do not know 16 (6%) Not in a relationship 125 (50%) The study instrument included the Beck Depression Inventory-II and interviewer-administered questionnaire.	Reproductive desires and intentions 66% (*n* = 166) of men and people under 40 wanted to have children. The best indicator of fertility desire and reproductive intentions among those who were currently in a relationship (*n* = 126) was not having a prior child. More serodiscordant (56%) individuals desired a child (*n* = 45) Depression More persons in serodiscordant (65%) compared to seroconcordant relationships (35%) reported depressive symptoms (*p* = 0.013) Awareness of assisted conception About 55% of the participants were aware that some fertility treatments with reduced transmission risk were available. Independent predictors of fertility motivation and desire The only independent predictor for fertility desire (*p* = 0.02) was ‘not having prior children. Independent predictors of depression Depressive symptoms were strongly predicted by the partner’s HIV status as well as the length of the diagnosis.	
2	Erhabor et al., 2012 (33)	Nigeria	To assess the reproductive health concerns among persons living with HIV/AIDS	Cross-sectional study 195 persons living with HIV	The following are the primary reproductive concerns: Transmitting the disease to a negative partner or unborn child (*n* = 48) 57.1% Passing away and leaving orphans behind (*n* = 24) 28.6% The inability of financial support in case of getting seriously ill (*n* = 12) 14.3%	
				people of reproductive age: 18–58 years (all subjects were < 58 years) Men: 88 Women: 107 The study instrument included a prevalidated, 16 structured interviewer-administered anonymous questionnaire with both open-ended and close-ended questions to collect quantitative data on baseline characteristics and reproductive health concerns.		
3	Saraswat et al., 2019 (11)	India	To describe the social, sexual, and reproductive issues and their impact on serodiscordant couples	Cross-sectional study 64 HIV serodiscordant couples were included in the study. 62 males were seropositive compared to 2 females. 61 patients (95.3%) were married, and 3 (4.6%) were unmarried. 36 patients (56.2%) were between the age group of 21 and 35 years, 21 (32.8%) between 36 and 55 years, and 7 (10.9%) between 56 and 70 years.	Impacts on sexual relationship and intimacy: Deciding for separation after diagnosis of disease (*n* = 11) 17.1% Relationship under constant strain (*n* = 31) 48.4% Post-diagnosis negative impacts on intimacy (*n* = 37) 57.8% Abstinence (*n* = 17) 26.5% Employing safe sex Practices (*n* = 16) 25% Difficulties that sexual partners face: Fear of infection transmission (*n* = 39) 60.9% Fear of pregnancy (*n* = 26) 40.6% Actual or perceived infidelity (*n* = 17) 26.5% Difficulties associated with condom use (*n* = 29) 45.3% Disinterest in the partner (*n* = 18) 28.1% Social problems (*n* = 17) 26.5% Knowledge of safer sexual practices (*n* = 12) 18% Social issues of HIV diagnosis: Deprivation of family/friend’s support (*n* = 26) 40.6% Disinterested family, friends, or neighbors (*n* = 19) 29.6% Satisfied with social support (*n* = 8) 12.5% Isolated in social meetings (*n* = 12) 18.7% Constantly probed to know the source of infection (*n* = 11) 17.1% Constant criticism that causes feelings of guilt or unworthiness (*n* = 8) 12.5% Social alienation of children at school (*n* = 13) 20.3% Social isolation and feeling of looniness at the workplace (*n* = 7) 10.9% Challenges related to reproduction: Established a family Yes: 49 (76.6%), No: 15 (23.4%) Intention to have children Yes: 41 (64.1%), No: 23 (35.9%) Reason to abstain from getting pregnant (Fear of infected child: 34 (53.1%), Social stigma: 22 (34.3%), Social alienation of the child: 29 (45.3%), Uncertainty of life: 16 (25%), Feeling of guilt: 19 (29.6%), currently pregnant: 1 (1.65%) Information about safer methods of conception Yes: 9 (14.1%), No: 55 (85.9%)	
4	Beckerman et al., 2002 (34)	America	To ascertain the central emotional challenges facing couples of mixed HIV status	Exploratory study The sample was collected from 11 sites comprising three outpatient hospitals and eight community-based AIDS support organizations. 150 questionnaires were distributed. 100 went directly to HIV-positive clients attending these sites half of this sample approved their partner’s involvement, and subsequently, 50 questionnaires were mailed to matched HIV-negative partners. A pilot instrument was built on themes identified in the existing literature within serodiscordant couples. Several interviews with experts who provide couple counseling to serodiscordant couples were held.	HIV transmission anxiety: 83% (*n* = 68) reported vital concern about HIV transmission 87% (*n* = 27) of women and 79% (*n* = 40) of men respondents identified fear of HIV transmission as a primary concern coping with the unpredictability of severe illness: 74% (*n* = 61) identified uncertainty as a primary emotional challenge. Changes in emotional relationship 76% (*n* = 62) report their time together as a couple as being more highly valued because of the impact of HIV on their lives. Consistent with this finding, 72% (*n* = 59) report their commitment to each other is stronger because of HIV in their life. Reproductive issues (the topic was asked about in the open-ended section): Fear of transmitting the virus and the probability of disabling illness in the future that interferes with parenting responsibilities were reported as the main barriers to establishing a family	
5	Nnebue et al., 2017 (35)	Nigeria	To compare the sexual and reproductive health needs and practices of HIV-discordant and-concordant couples	Cross-sectional study 289 (148 HIV-concordant and 141 HIV-discordant) couples were selected using a multistage sampling technique Quantitative data were collected by interview using a semi-structured questionnaire. Four focus group discussion sessions involving 10 participants	More concordant couples had sexual activity 142 (49.3%), in comparison to 122 (42.4%) of their discordant counterparts (*p* = 0.001) More discordant couples discussed sexual concerns (*p* = 0.05) After disclosing HIV diagnosis, 74 (26.3%) concordant and 68 (23.5%) discordant couples had children; while. Seventy-seven (26.6%) concordant couples desire to have children compared to 70 (24.2%) discordant ones Eight (2.8%) concordant couples and none of the discordant couples knew about artificial methods of conception.	
6	Scherer et al., 2014 (36)	America	To assess care providers for people living with HIV regarding attitudes, knowledge, and practice patterns toward fertility and conception in serodiscordant couples	Cross-sectional study 145 participants completed the survey Questions included: Demographic information of providers, attitudes towards the concept of reproduction, concept in PLWH (people living with HIV), knowledge of guidelines and practice patterns	Awareness of assisted conception: Only 3% reported being “very” familiar with the data on the safety of sperm washing with IUI, with 17% “somewhat” familiar, 24% “slightly” familiar, and 55% “not at all” familiar Sexual Relation: 38% percent reported having counseled serodiscordant couples on timed, unprotected intercourse without PrEP. Twenty-six percent reported being “very” familiar with the data on HIV transmission in serodiscordant couples, with 38% “somewhat” familiar, 20% “slightly” familiar, and 15% “not at all” familiar.	
7	Tchakounté et al., 2020 (37)	Cameroon	To describe the influence of HIV on sexual relationships and the decision to procreate	Cross-sectional study 346 couples were contacted: 192 HIV serodiscordant, 74 HIV positive seroconcordant, and 80 HIV seronegative concordant couples A self-administered questionnaire was used to collect social and demographic information.	Intimacy and sexual relations in a serodiscordant couple: Reduced frequency of sexual intercourses due to the discordance status (33.96%) Adverse effects on intimacy (43/39%) Condom use was not systematically (20/75%) The desire for childbearing in a serodiscordant couple: Did not want (additional) child/children (30/18%) Wanted (additional) child/children (69/80%)	
8	Rispel et al., 2011 (38)	South Africa and Tanzania	To investigate the influence of HIV on sexual relations and childbearing decisions of HIV-discordant couples	Exploratory study 26 discordant couples in South Africa and 10 couples in Tanzania were recruited. Self-administered questionnaires were used to obtain social and demographic information, while couples’ sexual relations and childbearing decisions were explored through in-depth, semi-structured individual and couple interviews. The study was limited to HIV-discordant couples, regardless of sexual orientation, who had been in a sexual relationship and were aware of the HIV-positive partner’s status for at least a year.	HIV-discordance, intimacy, and sexual relations:	
					South Africa (*n* = 52)	Tanzania (*n* = 20)
					*Tension due to discordant status:*	
					14/48	10/20
					*Intimacy affected:*	
					26/47	14/19
					*Safe sex*	
					37/48	12/20
					The desire for children and reproductive decisions:	
					With children (*n* = 44)	Without children (*n* = 23)
					Did not want: 26	Did not want: 6
					Wanted: 16	Wanted: 17
					Pregnant: 2	Pregnant: 0

### 3.1 Results of qualitative studies

#### 3.1.1 Childbearing intention

Two out of ten qualitative studies explored the childbearing intentions of the couples. A study identified several factors contributing to relinquished fertility intentions among HIV-discordant couples, including the risk of HIV vertical transmission during pregnancy, risk of sexual transmission to the uninfected partner, uncertainty about the future, and socioeconomic challenges (13). Another study reported that according to the experience of pregnancy, some couples stated that getting pregnant is an expected fact and pregnancy is a part of their life. The thought of having a child surprises them, even in the case of having children from previous relationships, but given what they have heard about HIV and discord, they thought they could not have children (29).

#### 3.1.2 HIV serodiscordance and sexual relations

Four articles out of ten qualitative articles dealt with issues related to the sexual relationship of these couples. There are many ideas about sexual behavior among these couples. A study revealed a spectrum of sexual behaviors among HIV-discordant couples, ranging from adherence to restrictive risk-reduction strategies to engagement in high-risk behaviors (23). The results of three studies showed that preventive strategies include low-risk sexual situations, PrEP,^3^ mutual masturbation, barriers (condoms), natural contraception (withdrawal), abstinence, and nonsexual ways to achieve intimacy (23, 26, 27). One study found that a subset of participants achieved risk reduction through consistent condom use. Some patients acknowledged risk reduction by relying on their partner’s highly active antiretroviral therapy (HAART) and undetectable viral load (i.e., TasP) and thought that immunity had developed after years of HIV exposure. Some believed that they definitely would use PrEP if they became familiar with PrEP at the same time as they started dating, but it is too late to accept them. This study found that a majority of participants lacked sufficient knowledge about the importance and accessibility of PrEP at their healthcare facility (23). A study revealed some views strongly prohibited discordant couples from any sexual contact. Such as: “In the process of continuing sexual activity, the negative person becomes infected. We can help by giving medicine to reduce the man’s desire. He does not crave, but his life continues” (25). It was reported in another study most of the couples reduced the frequency of sexual intercourse after their positive diagnosis, following some of the education they received with awareness of their condition. This study identified condoms as the most commonly used contraceptive method among the couples. Gender and discordant status were the most important determining factors for condom preference. The main problem of condom use was the agreement between the couple and continuous use (26). In another study, negotiations about sexual relationships were the most significant challenge that discordant couples reported. This study found that a majority of HIV-positive individuals in discordant relationships reported a complete loss of sexual interest. Condoms emerged as the most prevalent and preferred method of contraception among these couples (27).

#### 3.1.3 The need for psychological and social support

Four out of ten qualitative studies addressed the issue of psychological and social support. In three studies, it was reported that dealing with the discordance of serological status is one of the significant psychological distresses that these patients experience. In discordant couples, there is no ongoing involvement of the seronegative partner in the care process, and the main focus is on preventing HIV transmission, while their uninfected partner often struggles with many personal health issues that are inadequately addressed by their partner or HCPs.^4^ They not only suffer from various physical health-related issues, but they also consistently experience unpleasant emotions such as anger, sadness, hopelessness, depressive symptoms, anxiety, and fear. The obtained data show that proper counseling and psychological support of seronegative people are not implemented in these relationships (13, 23, 24). Also, one study reported that many who disclose report experiencing great anxiety, either because of HIV-related stigma or fear of losing the relationship (24). Another study reported that social rejection, the unstable physical health of a seropositive person, not paying attention to the needs of a seronegative spouse and children, and the inability to function as a parent can affect their physical and mental health (13). A study also reported that a positive HIV test makes discordant couples feel isolated. The absence of sufficient information regarding the condition may lead to rejection by couples’ social circles, as evidenced by previous studies (27).

#### 3.1.4 Need for training and consulting services

Six out of ten qualitative studies showed that these couples need training and consulting services. Four studies reported that in HIV-discordant relationships, there is an urgent need for education and counseling about the meaning of discordance, possible sources of infection transmission, and the window period to facilitate disclosure of HIV test results to the unaffected partner (13, 26–28). A study also showed that many people have a vague understanding of the serodiscordance situation and lack detailed knowledge about it. Upon inquiry regarding the discordant test results, a significant number of participants expressed confusion and disbelief, indicating a lack of trust in the test’s accuracy. The study also identified a high prevalence of concerns regarding both mortality associated with the partner’s condition and the participants’ long-term morbidity (26). One study highlighted the potential influence of HIV diagnosis timing on the need for counseling services. The study investigated whether the partner became aware of the serodiscordant status during an existing relationship or at its outset (27). In one study, the difference in the need for counseling was emphasized based on the time of HIV diagnosis (did the partner become aware of serodiscordance during the relationship or were they aware of it from the beginning of the relationship?) (24). Also, in a study, the need to create support group meetings for these couples, which provide comfort through understanding the previous experiences of others, has been shown (25).

#### 3.1.5 Access to reliable sources of information and correction of misconceptions

Four out of ten qualitative studies addressed the issue of reliable sources of information. Two studies reported that despite the extensive need for information and education about discordance to make crucial decisions about health and relationships, accessing evidence-based information for these couples is challenging (13, 30). A study also showed that two partners in this relationship have different learning and information needs. Unawareness of HIV discordance can lead to myths and misconceptions, including the belief that the uninfected partner must be infected, that the immune system of some people is resistant to HIV infection, or that infection cannot be transmitted in the case of mild sex. On the other hand, many people believed in pseudo-scientific explanations for HIV resistance, such as blood type and blood “strength” (26). One study additionally documented the presence of misconceptions among some participants, including beliefs in undetectable occult HIV infections and divine protection from acquiring HIV (27).

#### 3.1.6 The need for focused training to provide professional services

Four out of ten qualitative studies mentioned the issue of providing professional services to these couples. A study identified relationship management for serodiscordant couples seeking conception as a crucial consideration for healthcare providers, alongside clinical problem management. This study reported that healthcare providers do not discuss the couple’s pregnancy plans and fertility desires and refer discordant couples to specialist services for counseling rather than providing safer pregnancy guidance themselves. Some healthcare providers say there are no clear guidelines on what to recommend, and they have not been formally trained. Also, some providers are not comfortable talking about the sexual issues of these couples because they have not received enough training (28). In one study, one of the common complaints of these patients was the lack of human relations and discrimination by health care providers. When these people go to these centers they talk about their serological status, they face the violent behavior of the medical staff, and this causes these people to refuse to disclose their serological status in other situations (13). Also, the importance of evaluating serodiscordant couples’ relationship dynamics by healthcare providers was emphasized in two studies (23, 31). This evaluation can provide deeper insight to facilitate effective couples counseling.

### 3.2 Results of quantitative studies

Quantitative studies also support the results obtained in qualitative studies and overlap with them.

#### 3.2.1 Fertility desire

Pottinger and Carroll (32) reported in a cross-sectional study on 251 people with HIV (PLHIV), 26% of whom were serodiscordant couples, showed that people who are in serodiscordant relationships are more likely to have children than people who are in seroconcordant relationships. The study found a strong desire for parenthood among participants, with 66% (*n* = 166) expressing this interest. This desire was particularly prevalent in males and individuals younger than 40 years of age with no prior children (32). In a 2012 cross-sectional study of 195 people living with HIV (PLHIV), over half (56.9%) said they wanted to have children. Of the 111 subjects who indicated their desire to have children, women were more inclined to have children (64.5%) than men (47.7%) (33). A cross-sectional study that investigated sexual and reproductive challenges among 64 serodiscordant couples (2019) reported that 41 (64%) patients expressed the desire to have children as compared to 23 (35.9%). 26 (40.6%) patients had a fear of pregnancy and its outcome (11). A cross-sectional study by Beckerman (34), in 150 discordant couples, showed that more than half the respondents commented that with fear of passing infection to a child as well as the fear that one of them would be too ill to parent, HIV was seen as an unyielding obstacle to starting a family. Nnebue et al. (35) conducted a cross-sectional study on the reproductive health needs of 289 couples (148 HIV-concordant and 141 HIV-discordant). They found no statistically significant difference (*p* = 0.255) in the proportion of couples with children after HIV diagnosis: 74 (26.3%) of concordant couples and 68 (23.5%) of discordant couples. Similarly, no significant difference (*p* = 0.731) was observed in the desire for children; 77 (26.6%) of concordant couples and 70 (24.2%) of discordant couples wanted to have children (35). Tchakounté et al. (37), in a cross-sectional study on 53 serodiscordant couples showed that 37 out of 53 (69.8%) HIV serodiscordant couples wanted children, among them, seven couples did not have any and expressed their aspiration for parenthood despite fear of infecting one’s partner (37). Rispel et al. (38), in a cross-sectional study on 36 HIV-discordant couples, showed that 17 out of 23 childless participants and 16 out of 44 participants who had a previous child wanted another child. However, the desire was influenced by fear of HIV transmission to the negative partner and medical professional advice (38).

#### 3.2.2 Sexual relations

A 2019 cross-sectional study investigating sexual and reproductive health challenges among 64 serodiscordant couples found that 39 (60.9%) of participants reported a history of fearing disease transmission to their seronegative partner. This fear was shared by both partners. 29 (45.3%) patients complained of issues due to condom usage regarding the lack of spontaneity or satisfaction. 18 (28.1%) patients admitted to having lost interest in their spouses after they were detected seropositive. Only 12 (18%) couples were aware of the safer sexual practices. 55 (85.9%) couples did not know safe conception methods, while 9 (14.1%) had satisfactory knowledge of the same (11). A 2002 cross-sectional study by Beckerman involving 150 discordant couples found that 83% (*n* = 68) of participants reported strong concern about HIV transmission within their relationship (34). Nnebue et al. (35), in a cross-sectional study of the reproductive health needs of 289 (148 HIV-concordant and 141 HIV-discordant) couples, stated that more concordant couples 142 (49.3%), compared to 122 (42.4%) discordant counterparts were sexually active (*p* = 0.001). More discordant couples had sexual concerns to share (*p* = 0.05). Only 9 (3.2%) concordant couples, compared to 3 (1.1%) discordant couples, had good knowledge of family planning methods by at least one of the partners (*p* = 0.097). 131 (48.3%) concordant couples used condoms more frequently than the 119 (43.9%) discordant couples. More concordant couples (29 [10%]) than discordant couples (9 [6.6%]), had good knowledge of emergency contraception (*p* = 0.047) (35). A 2015 cross-sectional study by Scherer et al. involving 145 HIV service providers in New York City, USA, found that 38% reported counseling serodiscordant couples about timed, unprotected intercourse without pre-exposure prophylaxis (PrEP). Twenty six percent reported being “very” familiar with the data on HIV transmission in serodiscordant couples, with 38% “somewhat” familiar, 20% “slightly” familiar, and 15% “not at all” familiar (36). Tchakounté et al. (37) conducted a cross-sectional study on 53 serodiscordant couples. They found that 33.96% of couples reported experiencing tensions related to HIV serodiscordance after learning their HIV statuses. This was evidenced by a reduction in sexual frequency. This study found that serodiscordant couples reported having sexual desire disorders at a rate of 43.39%. The systematic use of condoms (male only) was mainly reported among 20.75% of serodiscordant couples. Among serodiscordant couples, 18.86% reported never using condoms, while 60.37% indicated occasional condom use (37). Rispel et al. (38) conducted a cross-sectional study on 36 HIV-discordant couples. They found that 24 out of 68 respondents (35.3%) reported experiencing tension in their relationship, and 40 out of 66 (60.6%) reported that intimacy was affected by their discordant status. Additionally, 49 out of 68 participants (72.1%) engaged in safe sex. A study has identified several problems associated with condom use: loss of spontaneity, decreased sexual desire and frequency of intercourse, avoidance by one partner, and conflict between parenthood intentions and HIV transmission prevention (38).

#### 3.2.3 Awareness of assisted conception

A 2019 cross-sectional study involving 251 people living with HIV (PLHIV), including 26% serodiscordant couples, investigated awareness of fertility treatments for reducing transmission risk. The study found that slightly more than half (55%) of the participants were aware of these fertility options (32). A cross-sectional study (2012) conducted on 195 people living with HIV (PLHIV) showed that only 58% of the serodiscordant couples had received reproductive health counseling from HIV counselors. The reasons for not seeking advice were anticipated adverse reactions and discrimination from the counselors. The majority of subjects were only aware of some reproductive health options available to reduce the risk of infecting their partners and/or baby, such as artificial vaginal insemination, intrauterine insemination, cesarean section, avoidance of breastfeeding, and prenatal pre-exposure prophylaxis for the fetus. They were unaware of other options, such as sperm washing, *in vitro* fertilization, and intracytoplasmic sperm injection (33). Nnebue et al. (35), in a cross-sectional study of the reproductive health needs of 289 (148 HIV-concordant and 141 HIV-discordant) couples, stated that 114 (39.9%) concordant couples compared to 104 (36.4%) discordant couples had more awareness of artificial conception methods. Among concordant couples, 8 (2.8%) practiced artificial conception methods, compared to none in discordant couples (*p* = 0.007). Concordant couples were less likely to use artificial conception methods due to perceiving them as unnecessary, whereas discordant couples did not use them due to lack of knowledge (*p* = 0.01) (35). A cross-sectional study by Scherer et al. (36) on 145 HIV service providers in the USA in New York City showed that Only 3% reported being “very” familiar with the data on the safety of sperm washing with IUI, with 17% “somewhat” familiar, 24% “slightly” familiar, and 55% “not at all” familiar.

#### 3.2.4 Family and social status

A cross-sectional study that investigated sexual and reproductive challenges among 64 serodiscordant couples (2019) reported that over 26 (40.6%) of couples did not receive family support, while nearly 19 (29.6%) reported family indifference regarding the seropositive status. 12 (18.7%) couples experienced isolation in public gatherings, while 11 (17.1%) said they were constantly probed by people regarding the source of infection. 13 (20.3%) couples said that their children were isolated in school due to the seropositive status of the parent, and 8 (12.5%) had a feeling of guilt and worthlessness resulting from the behavior of society toward the patients and their family. More than 7 (10.9%) of couples reported workplace isolation, while satisfaction with social support from friends, family, and society was low 8 (12.5%). 17 (26.5%) seronegative partners had issues because of actual or perceived infidelity of the HIV-positive partner (11). A cross-sectional study by Beckerman (34), in 150 discordant couples, showed that 74% (*n* = 61) identified uncertainty as a primary emotional challenge. 76% (*n* = 62) report their time together as a couple as being more highly valued because of the impact of HIV on their lives. Consistent with this finding, 72% (*n* = 59) report their commitment to each other is stronger because of HIV in their life (34). A cross-sectional study (2019) of 251 people living with HIV (PLHIV), including 26% in serodiscordant couples, found a significantly higher prevalence of depressive symptoms in the serodiscordant group (65%) compared to the seroconcordant group (35%) (*p* = 0.013). Further, PLHIVs in their reproductive years who are at depression risk are those in a serodiscordant relationship (p = 0.01) and who have been diagnosed between 1 and 4 years (*p* = 0.05) (32).

## 4 Discussion

HIV-discordant couples, just like the other couples, have various reproductive health needs that must be met, such as safe sexual intercourse, family planning, contraception, infertility treatments, and so on. We discussed the findings of existing quantitative and qualitative pieces of evidence in this section.

The results of one systematic review and meta-analysis suggest that many individuals in HIV-serodiscordant relationships have fertility desires/intentions, although the prevalence is particularly heterogeneous in LMIC (low-and middle-income countries) in comparison to HIC (high-income countries) (39). Also in other studies, the prevalence of reproductive desire in HIV serodiscordant couples was relatively high (40). Our findings indicate that childbearing intentions in HIV-discordant couples are comparable with the findings of this study. It has been reported that some of the influential factors on fertility intentions of HIV serodiscordant couples were: age (41), number of children (39, 42, 43), having a partner who wanted children (39, 42), religion (41), discussions with health workers (39) and maintaining the stability of the union and sociocultural pressures (43).

Sexual desire and interest can be negatively affected by fear of infection transmission to an uninfected partner, guilt and shame due to HIV-associated stigma, or psycho-emotional distress. Concomitant with improvements in the health status of individuals living with HIV and the increased availability of antiretroviral therapies, there may be a growing interest in sexual relationships among men and women with HIV (44). Couples seem to support continued sexual activity in a discordant relationship, but there is consensus on the need to adopt safer sex practices. Continued sexual activity is essential to maintain the relationship (25). The fear of transmitting the disease is one of the main issues that these couples face, which negatively affects their sex life (11). The risk of HIV transmission to a seronegative partner depends on the viral load, frequency, and type of sexual activity (45, 46). As of September 2015, WHO recommends that people at substantial risk of HIV infection should be offered tenofovir disoproxil fumarate (TDF)-based oral PrEP as an additional prevention choice as part of comprehensive prevention. Oral PrEP is highly effective at preventing HIV when used as directed (47). There are questions regarding the long-term effectiveness of condoms (48). A WHO study in six African countries found that consistent motivational support from healthcare providers (HCPs) can significantly increase awareness of and facilitate condom application among HIV-positive individuals (49). Therefore, educational programs emphasizing the importance and effectiveness of condom use should be considered an integral component of post-test counseling, education, and support services provided to HIV-positive couples (50, 51). This review identified heterogeneity in condom use among couples across the included studies. The observed heterogeneity in condom use among couples likely stems from variations in study design, sample size, and social, demographic, economic, and cultural factors specific to each included country or region. These findings emphasize the importance of couple-oriented HIV prevention services, treatment as prevention (TasP), and sexual health services tailored to improve the quality of life for HIV-serodiscordant couples (52).

HIV-discordant couples generally experience a mild to moderate degree of psychological distress, compared to the general population. In contrast, couples who practice open communication tend to report lower levels of distress (53). One study found that HIV-positive and HIV-negative partners in serodiscordant relationships can experience psychosocial and emotional distress symptoms, including anger, blame, desperation, depression, anxiety, and even suicidal ideation in some cases (54). Research suggests that incorporating psychological and social support into post-test counseling sessions can effectively improve general well-being, disease management, and relationship dynamics among HIV-positive couples (52, 55). Psychosocial support is vital in disease prevention, health promotion, treatment compliance, and recovery (56). Screening for depression is particularly recommended for discordant couples in their reproductive years who have been diagnosed with HIV for more than a year and who are experiencing depressive symptoms. In addition, clinicians should be aware of emotional distress symptoms such as guilt, depression, suicidal thoughts, and loss of libido since these symptoms are frequently found between couples who desire to have children.

Counseling and providing in-depth educational information at every stage of the treatment process is crucial to help couples and provide them with the necessary information (57). The findings of a study showed that considering regular counseling sessions for serodiscordant couples is a highly effective prevention strategy and can consequently lead to better clinical outcomes (58). Counseling can also be a significant influence in addressing the psychological needs of HIV-discordant couples (59). Sexual HIV prevention counseling and guidance for people in discordant relationships is an essential part of HIV care (60). They also emphasize the importance of psychosocial counseling and support in helping couples cope with the emotional consequences of serodiscordance and disclosure (61).

Discordant couples require access to current, evidence-based information on HIV, AIDS, and serodiscordance to make informed decisions about their health and relationships. However, our study found that a majority of these patients reported difficulty obtaining such resources and encountered social stigma and misconceptions. Our findings differ from previous studies conducted in Asian, African, and Latin American contexts, which suggested that media-provided information can be effective in preventing HIV transmission (62). The spread of misinformation can fuel stigma, lead to the inaccurate application of prevention methods, and consequently, increase the risk of seroconversion (63). In the absence of a widely available and effective HIV vaccine, access to accurate information remains one of the most effective tools for controlling the HIV epidemic. Adequate informative interventions can potentially change people’s behavior and reduce HIV incidence (64).

Empowering people living with HIV to make informed decisions about their reproductive health is a strong ethical imperative to support their sexual and reproductive health needs. Findings suggest that some HCPs may avoid contact with discordant couples and instead refer them to specialized centers. The improvement and availability of safer pregnancy services for all HIV-infected couples depend on adequate education for healthcare providers at all levels regarding safe pregnancy strategies for serodiscordant couples (65). While research suggests limited involvement of healthcare providers (HCPs) in the pregnancy planning and reproductive intentions of discordant couples, studies directly exploring HCP perspectives on this topic remain scarce (66–69). Effective safer conception services for HIV-discordant couples require a couple-centered approach and rely on healthcare providers’ (HCPs) ability to concurrently manage clinical aspects alongside the gendered and relational needs of individuals within the relationship (70–72). It is also recommended that all HPCs should be aware of gendered vulnerabilities, the risk of intimate partner violence (IPV), and cultural beliefs and norms about fertility and pregnancy (28). Reproductive and sexual health care for HIV-infected women should include integral programs for screening lower genital tract and sexually transmitted diseases, evaluation of contraceptive options, and risk-reduction and preconception counseling (73). HCPs should also be educated about IPV, relationship dynamics, the emotional consequences of disclosure, and the need for couple support (74).

## 5 Conclusion

This study highlights the various challenges faced by HIV-discordant couples in meeting their reproductive health needs. Unfortunately, most HIV prevention, care, and treatment programs lack well-designed protocols to adequately address the unique health needs of HIV-discordant couples. Life prolongation without considering the basic human needs of these groups is the worst policy to deal with this disease. Policymakers, program implementers, and clinicians should pay more attention to these groups. Developing comprehensive guidelines for reproductive and sexual health, psychological rehabilitation, and fertility planning tailored to serodiscordant couples is crucial for improving their quality of life and health outcomes.

## Data Availability

The raw data supporting the conclusions of this article will be made available by the authors, without undue reservation.

## Footnotes

^1^Sexual and reproductive health.

^2^ www.opengrey.eu

^3^Pre-Exposure Prophylaxis.

^4^Healthcare providers.
