# Supplementary material for: Reproductive health needs of HIV serodiscordant couples: a systematic review
Source: Front Public Health. 2024 Aug 29;12:1348026. doi: 10.3389/fpubh.2024.1348026 (PMC11390637; doi:10.3389/fpubh.2024.1348026)
Supplement: Supplementary file 1 [file Table_1.DOC]

**Search Strategy in databases**

**PubMed:** (((((((((((((((AIDS) OR (Human Immunodeficiency Virus[Title/Abstract])) OR (Immunodeficiency Virus, Human[Title/Abstract])) OR (Immunodeficiency Viruses, Human[Title/Abstract])) OR (Virus, Human Immunodeficiency[Title/Abstract])) OR (Viruses, Human Immunodeficiency[Title/Abstract])) OR (Human Immunodeficiency Viruses[Title/Abstract])) OR (AIDS Virus[Title/Abstract])) OR (AIDS Viruses[Title/Abstract])) OR (Virus, AIDS[Title/Abstract])) OR (Viruses, AIDS[Title/Abstract])) AND (Need[Title/Abstract])) OR (Needs[Title/Abstract])) OR (information[Title/Abstract])) AND (couple[Title/Abstract])) AND (Serodiscordant[Title/Abstract])

((((((((((((((((AIDS) OR (Human Immunodeficiency Virus[Title/Abstract])) OR (Immunodeficiency Virus, Human[Title/Abstract])) OR (Immunodeficiency Viruses, Human[Title/Abstract])) OR (Virus, Human Immunodeficiency[Title/Abstract])) OR (Viruses, Human Immunodeficiency[Title/Abstract])) OR (Human Immunodeficiency Viruses[Title/Abstract])) OR (AIDS Virus[Title/Abstract])) OR (AIDS Viruses[Title/Abstract])) OR (Virus, AIDS[Title/Abstract])) OR (Viruses, AIDS[Title/Abstract])) AND (Need[Title/Abstract])) OR (Needs[Title/Abstract])) OR (information[Title/Abstract])) AND (couple[Title/Abstract])) AND (Serodiscordant[Title/Abstract])) AND (Reproductive health[Title/Abstract])

**Scopus:** ALL (AIDS OR Human Immunodeficiency Virus OR Immunodeficiency Virus Human OR Virus Human Immunodeficiency OR Viruses Human Immunodeficiency OR Human Immunodeficiency Viruses OR AIDS Virus OR AIDS Viruses OR Virus AIDS AND Need OR Needs OR information AND couple AND Serodiscordant)

ALL (AIDS OR Human Immunodeficiency Virus OR Immunodeficiency Virus Human OR Virus Human Immunodeficiency OR Viruses Human Immunodeficiency OR Human Immunodeficiency Viruses OR AIDS Virus OR AIDS Viruses OR Virus AIDS AND Need OR Needs OR information AND couple AND Serodiscordant AND Reproductive Health)

**Web of science:** (aids OR human AND immunodeficiency AND virus OR immunodeficiency AND virus, AND human OR immunodeficiency AND viruses, AND human OR virus, AND human AND immunodeficiency OR viruses, AND human AND immunodeficiency OR human AND immunodeficiency AND viruses OR aids AND virus OR aids AND viruses OR virus, AND aids OR viruses, AND aids AND need OR needs OR information AND couple AND serodiscordant)

(aids OR human AND immunodeficiency AND virus OR immunodeficiency AND virus, AND human OR immunodeficiency AND viruses, AND human OR virus, AND human AND immunodeficiency OR viruses, AND human AND immunodeficiency OR human AND immunodeficiency AND viruses OR aids AND virus OR aids AND viruses OR virus, AND aids OR viruses, AND aids AND need OR needs OR information AND couple AND serodiscordant AND Reproductive Health)

**Google scholar:** “HIV” OR “AIDS” AND “Reproductive Health” OR “Reproductive” AND "Health" AND “Sexual Health” OR "Sexual" AND "Health" AND “Need” OR “Needs” OR “Demand” OR "Demands" AND “Serodiscordant Couple” OR “Serodiscordant” OR “Couple”
